# Nonfunctional Metastatic Parathyroid Carcinoma in the Setting of Multiple Endocrine Neoplasia Type 2A Syndrome

**DOI:** 10.1155/2014/731481

**Published:** 2014-02-20

**Authors:** María Posada-González, Joaquín Gómez-Ramírez, Manuel Luque-Ramírez, Mercedes Guijarro, Elena Martín-Pérez, Ana Rodríguez-Sánchez, Iñigo García-Sanz, Eduardo Larrañaga

**Affiliations:** ^1^Department of General and Gastrointestinal Surgery, La Princesa University Hospital, 62 Diego de Leon Street, 28006 Madrid, Spain; ^2^Department of General and Gastrointestinal Surgery, Fundación Jiménez Díaz Hospital, 2 Reyes Catolicos Avenue, 28040 Madrid, Spain; ^3^Department of Endocrinology and Clinical Nutrition, Ramón y Cajal University Hospital, Colmenar Viejo Road 9.100 Km, 28034 Madrid, Spain; ^4^Department of Pathology, La Princesa University Hospital, 62 Diego de Leon Street, 28006 Madrid, Spain

## Abstract

Parathyroid carcinoma is a very rare malignancy. It has been associated with hyperparathyroidism-jaw tumour syndrome, familial isolated primary hyperparathyroidism, and multiple endocrine neoplasia type 1 (MEN-1) and 2A (MEN-2A) syndromes. We report a 54-year-old man with a MEN-2A which presents with a nonfunctional metastatic parathyroid carcinoma and a pheochromocytoma in the absence of medullary thyroid carcinoma. Only a few cases of parathyroid carcinoma have been reported in the literature associated with this syndrome.

## 1. Introduction

Multiple endocrine neoplasia type 2 (MEN-2A) is a hereditary syndrome characterized by the occurrence of two or more specific endocrine tumours (medullary thyroid carcinoma, pheochromocytoma, or parathyroid adenoma/hyperplasia). MEN-2A is an autosomal dominant disorder, but each clinical entity is expressed with variable degrees of penetrance. Almost 100% of patients with MEN-2A develop medullary thyroid carcinoma (MTC). The frequency of pheochromocytoma among these patients is 50% and of hyperparathyroidism only 10–30% [1].

Parathyroid carcinoma (PCA) is not a feature of multiple endocrine neoplasia syndromes. In the bibliographic search, only 7 cases of PCA have been described in the setting multiple endocrine neoplasia syndrome type 1 (MEN-1) [2]. To our knowledge, only two cases of parathyroid carcinoma have been reported in a MEN-2A [3, 4].

PCA is the rarest endocrine malignancy. More than 90% of PCA are functional, presenting with symptoms of hyperparathyroidism due to the effects of elevated serum parathormone (PTH) and calcium levels [5]. Nonfunctional PCA are extremely rare, with less than 20 reported cases since 1929 [6].

We present a very unusual case of nonfunctional PCA in the setting of a MEN-2A.

## 2. Case Presentation

We report a 54-year-old man with a past medical history of type 2 diabetes mellitus, obstructive sleep apnoea, and family history of multiple endocrine neoplasia type 2A. The genetic study revealed a heterozygous mutation (*Cys618Arg*) in exon 10 of the oncogene RET.

He did not have any symptoms and did not refer history of hypertension or paroxysmal hypertensive crises. The serum and urinary catecholamines remained normal. The T2-weighted magnetic resonance imaging showed two subcentimeter-sized lesions in the left adrenal gland, and the 123-I-metaiodobenzylguanidine (MIBG) scan confirmed a functional nodular lesion. A laparoscopic left adrenalectomy was performed, and the final pathology confirmed a pheochromocytoma.

**Figure 1:**
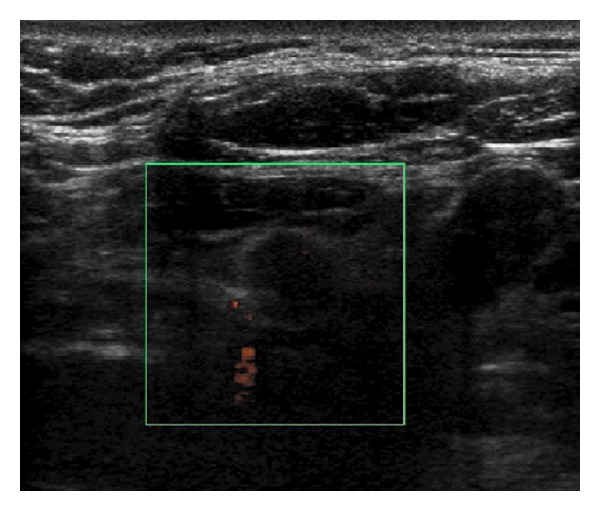
Thyroid ultrasound. Hypoechoic nodule in the inferior pole of the left thyroid lobe.

**Figure 2:**
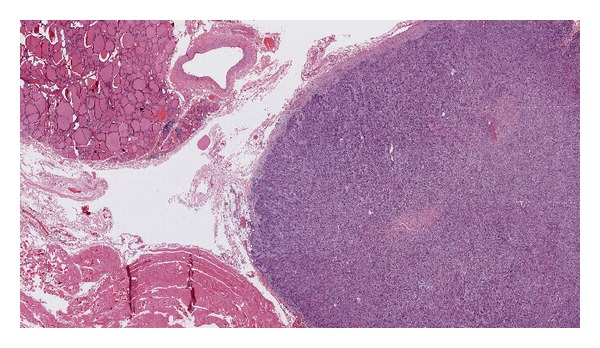
Tissue sample. A 1.4 cm irregularly shaped encapsulated nodular lesion in the inferior pole of the left thyroid lobe which did not depend on the thyroid parenchyma.

**Figure 3:**
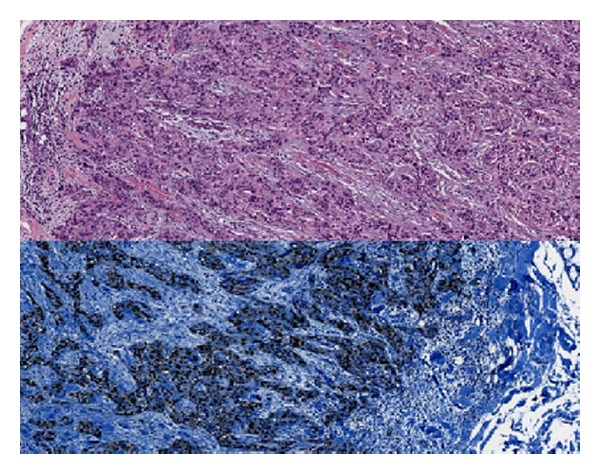
Tissue sample. The microscopic examination of the nodular lesion shows parathyroid chief cells in trabecular, infiltrative pattern with fibrotic septae and vascular invasion. Immunohistochemical analysis revealed a nuclear overexpression of cyclin D1. Cyclin D1 stains the nuclei blue.

**Figure 4:**
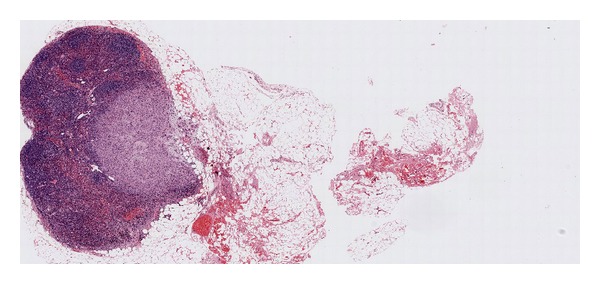
Tissue sample. Two millimetres lymph node metastasis with extracapsular invasion.

Laboratory tests showed normal calcitonin, calcium, and parathormone serum levels. The thyroid ultrasound showed a hypoechoic nodule in the inferior pole of the left thyroid lobe (Figure 1). The mutation (*Cys618Arg*) in exon 10 of the oncogene RET is described as a mutation with a higher risk of developing a medullary thyroid cancer, so a prophylactic total thyroidectomy with central lymphadenectomy was performed 3 months after adrenalectomy. The pathological examination showed a 1.4 cm irregularly shaped encapsulated nodular lesion in the inferior pole of the left thyroid lobe which did not depend on the thyroid parenchyma (Figure 2). In the microscopic examination, we found parathyroid chief cells in trabecular pattern with fibrotic septae, nuclear atypia, and vascular invasion. Immunohistochemical analysis revealed an overexpression of cyclin D1 (Figure 3). A two millimetres lymph node mestastasis with extracapsular invasion was also found (Figure 4). There was no evidence of medullary thyroid carcinoma.

## 3. Discussion

PCA are very infrequent tumors. With an estimated incidence of less than 1% in all cases surgically treated for primary hyperparathyroidism, they are one of the rarest of all human cancers. According to NCDB reports, their survival at five years is 85.5% and at ten years is 49.5%. The median age of presentation in most series is between 45 and 60 years. In contrast with primary hyperparathyroidism, which is more common in women (3-4 : 1), the incidence of PCA is equal between the two genders [5].

The exact aetiology of PCA remains unclear. PCA may appear as a sporadic disease although several genetic defects have also been reported to be involved. Some oncogenes and tumour suppressor genes have been associated to the pathogenesis of PCA. Studies on the tumour suppressor gene CDC73 (formerly HRPT2) have provided the best evidence [7]. CDC73 encodes a protein called parafibromin (parathyroid disease and fibro-osseous lesions). The parafibromin inhibits cyclin D1 activity promoting cellular apoptosis and avoiding cell proliferation. Some studies have shown that mutations of CDC73 may result in a loss of parafibromin expression and, as it is the case of our patient, cyclin D1 overexpression which promotes the neoplastic transformation of the parathyroid tissue [8]. However, more studies are needed to understand the exact role of CDC73 and parafibromin in the pathogenesis of PCA [9].

There are also some other clinical situations such as external radiation neck exposure or end-stage renal disease, which may predispose to the development of PCA. In addition, PCA has been associated with familial isolated primary hyperparathyroidism, hyperparathyroidism-jaw tumour syndrome, MEN-1, and MEN-2A [6].

Apart from our patient, there are just two cases of PCA in the setting of MEN-2A reported in the literature. Details of the cases are displayed in Table 1.

More than 90% of PCA are functional, presenting with symptoms of hyperparathyroidism due to effects of elevated serum parathormone (PTH) and calcium levels. Malignancy is generally related to calcium levels greater than 3.5 mmol/L and PTH levels more than 5 times the upper limit of the normal range. Nonfunctional PCA are extremely rare. They account for approximately 1.9% of parathyroid tumours with less than 20 reported cases since 1929 [6, 10]. As in our patient, nonfunctional PCA remain normocalcemic with normal serum levels of PTH.

Most of the reported normocalcemic PCA cases present with locally advanced neck masses. This may be due to the fact that they are larger at presentation because of the absence of clinical manifestations. They also show frequent locoregional metastasis and recurrence. Despite the fact of the short number of nonfunctional PCA cases described and the difficulty to compare them to patients with functional PCA, nonfunctional PCA seem to be more aggressive tumors representing a poor prognosis factor [10].

The treatment of PCA is based on the complete surgical removal of the tumors including adjacent tissues involved. This is the only curative treatment which provides the best long-term survival. Nonsurgical therapies such as chemotherapy and radiation have shown poor results and their use in the PCA treatment is still not well defined [9].

The diagnosis of PCA requires a histological confirmation. However, there is a big difficulty to distinguish between the histopathological features of benign and malignant parathyroid tumors. In 1973, Schantz and Castleman published the histological criteria for the diagnosis of PCA [11]. These classical histological features which define PCA are the presence of capsular or vascular invasion, trabeculated fibrous parenchyma, and mitotic figures. Unfortunately, these features can also appear in benign parathyroid tumours, so this is why the capsular or vascular invasion are considered to be the most specific features of PCA [12]. Some immunohistochemical markers have been used to try to improve accuracy in identifying parathyroid malignancy. As we have already indicated, overexpression of cyclin D1 seems to be a specific feature of PCA. As in our patient, it has been identified in a high percentage of parathyroid carcinomas. In 1999, Vasef et al. presented evidence that cyclin D1 is highly expressed in PCA specimens (91%) compared with parathyroid adenomas (39%) [13].

Our patient represents a very unusual case, and not only because of the presence of PCA associated to a MEN-2A syndrome. It is also remarkable that our patient shows no evidence of MTC, even though the penetrance of MTC in the MEN-2A syndrome is almost 100%. Nowadays and thanks to all studies developed during the last years, some mutation-specific risk profiles (genotype-phenotype correlation) have been established. The mutation of the RET proto-oncogene found in our patient (Cys618Arg) shows a high risk to develop a MTC [14]. However, the histopathological study after the thyroidectomy revealed no signs of MTC.

We must conclude that this patient may be representing a variation from the classical multiple endocrine neoplasia syndrome type 2A which develops a pheochromocytoma and a parathyroid carcinoma in the absence of medullary thyroid carcinoma.

## Figures and Tables

**Table 1:** Cases of PCA in the setting of MEN-2A reported in the literature.

Reference	Jenkins et al. (1997) [3]	J. J. Alfaro (2002) [4]	This case
Age (years)	47	49	54
Sex	Male	Male	Male
RET mutation	(Cys634Tyr) of exon 11	Not known mutation	(Cys618Arg) of exon 10
Clinical presentation	8-week symptoms of thirst and polyuria	Severe hypercalcemia	Asymptomatic
Serum calcium (mmol/L)∗	3.3	3.77	2.3
Serum PTH (pmol/L)°	102	1399	6.1
Serum calcitonin (ng/L)^6^	5110	75.8	2.1
Serum and urinary metanephrines	Normal	Normal	Normal
Tumour size (cm)	Not recorded	Not recorded	1.4
Metastases	First thoracic vertebra (T1) and right frontal bone	Lung	Cervical lymph node
Medullary carcinoma	Yes	Yes	No
Pheochromocytoma	No	No	Yes

*Serum calcium normal limit (2.1–2.5 mmol/L).

°Serum PTH normal limit (11–54 ng/L).

^6^Serum calcitonin normal limit (males < 13.8 ng/L; females < 6.4 ng/L).
